# Animal Models of Alzheimer's Disease

**DOI:** 10.4061/2010/606357

**Published:** 2011-06-29

**Authors:** Gemma Casadesus, Gary Arendash, Frank Laferla, Mike McDonald

**Affiliations:** ^1^Department of Neurosciences, School of Medicine, Case Western Reserve University, 2109 Adelbert Road, E-729, Cleveland, OH 44106, USA; ^2^Department of Cell Biology, Microbiology and Molecular Biology, University of South Florida, USA; ^3^School of Biological Sciences, Institute for Brain Aging and Dementia, Neurobiology and Behavior, UC Irvine, USA; ^4^Department of Neurology, The University of Tennessee Health Science Center, USA

The necessity for a deeper understanding of neurological diseases such as Alzheimer's disease (AD) that increase in frequency as a function of age has become of paramount importance with the coming of age of the baby boom generation and the increasing social demands for individuals to perform better and longer. AD is characterized by a gradual decline in cognitive function and presence of pathological inclusions such as A*β* plaques and neurofibrillary tangles composed of hyper-phosphorylated tau in the brain. These are the main hallmarks of the disease and focus of most current therapeutic strategies. As such, the development of transgenic and non-transgenic models of AD over the last decade has primarily focused on these pathological markers. These models have become promising tools to decipher the mechanistic importance of tau phosphorylation and A*β* deposits, as well the relationship between each other, other pathological AD-related events, and cognitive loss. However, while seemingly obvious, it is important to remember that the validity of an animal model of disease is tightly linked to the ability of the animal to mimic the signs of the disease—which goes beyond the pathology and needs to include cognitive decline and neuronal loss. This special issue seeks to provide an updated and critical evaluation of the available animal models of AD with the primary goal to deepen our mechanistic understanding of AD and elucidate how the development of these models has led or can lead to novel therapies for AD patients.

The topics included in this special issue are the following:

- Development of novel transgenic or non-transgenic animal models of AD (rodents or other).
- Mechanistic studies of AD-related pathology in animal models including but not limited to APP/A*β* and/or tau hyper-phosphorylation (i.e., oxidative stress, mitochondrial, inflammatory, cell cycle changes etc.)
- Development of novel behavioral or translational methodologies to determine impairment in models of AD.
- Novel therapeutic approaches to modulate AD pathology and cognitive impairment.

In the coming years, prevalence of Alzheimer's disease (AD) is said to overtake diseases such as AIDS or cardiovascular diseases (World Health Organization). The impact of this disease on millions of individuals, their families, and the health care system will be devastating. As such, the scientific community has strived to model AD in the hope that these models will provide the tools for effective and desperately needed therapeutic development and testing. At the forefront of the quest to decipher Alzheimer's disease are animal models. Developed through genetic, chemical, and/or lesions, animal models of AD try to faithfully mimic disease pathogenesis in a growing number of species ranging from invertebrates to higher mammals such as primates. The ultimate goal is to further our understanding of mechanisms associated with this plural and complex disease and allow us to test promising therapies to manage, prevent, and hopefully cure AD. 

Novel animal models of AD are relentlessly being developed and existing ones fine-tuned; however, they face the challenges associated with the complexity of a neurodegenerative disease. For example, most animal models of AD do not reproduce the full phenotypical disease spectrum. Also relevant is the fact that, like for most neurodegenerative diseases, the etiology of AD and the clinical presentation differs greatly across individuals. As such, while the current models are very well suited for the study of specific pathology-driven mechanisms, most notably amyloid-beta, pharmacological testing in animal models of neurodegenerative disease often translates into poor efficacy when applied to the clinical population. With these advances and challenges in mind, this special issue, written by experts in the field, provides a rich and updated overview of disease-related aspects modeled in several species, ranging from the established transgenic models but also including novel drosophila and chick models. A wide range of modeled disease-related events are discussed at all levels, from descriptive and mechanistic to technical in order to provide a full scope of this disease and additional techniques that may become useful to investigators in the field. 

An important aim of this special issue is to provide an accurate picture of the plurality of this disease by presenting research that is not only focused on modeling primary pathology. This includes, the downstream impact of tau phosphorylation in both murine and drosophila models and presenilin-mediated signaling, and also disease-related events that go beyond classical pathology. This special issue includes primary research and review articles on oxidative stress and mitochondrial dysfunction, lipid raft alterations in the murine models of the disease, and attention to various enzymatic complexes including the 2-oxoglutarate dehydrogenase complex. Furthermore, classic neurotransmitter systems such as acetylcholine, and brain regions such as the locus coeruleus are also discussed in an attempt to highlight the importance of these alternate or additional processes in the disease and provide a breath of how AD can be modeled at different levels. In conjunction with these articles, this issue also includes in depth discussion of biomarkers, imaging, and behavioral techniques, and studies presenting gender differences in murine and nonmurine models of AD that provide additional insight to investigators in the field.

Taken together, the ultimate outcome of this exciting and plural special issue is to enrich current research on this devastating disease with the hope that it will help to get us a step closer to a much needed cure. 

*Gemma Casadesus* *Gemma Casadesus* *Gary Arendash* *Gary Arendash* *Frank Laferla* *Frank Laferla* *Mike McDonald* *Mike McDonald*

